# Most patients with COPD are unaware of their health threats and are not diagnosed: a national-level study using pulmonary function test

**DOI:** 10.1038/s41598-023-32485-9

**Published:** 2023-04-11

**Authors:** Myung-Bae Park, Tae Sic Lee, Ji-ho Lee, Jinhee Lee

**Affiliations:** 1 Department of Health and Welfare, Pai Chai University, Daejeon, Republic of Korea; 2 Department of Family Medicine, Yonsei University Wonju College of Medicine, Wonju, Republic of Korea; 3 Department of Internal Medicine, Yonsei University Wonju College of Medicine, Ilsan-Ro, Wonju, 26426 Republic of Korea; 4 Department of Psychiatry, Yonsei University Wonju College of Medicine, 20 Ilsan-Ro, Wonju, 26426 Republic of Korea

**Keywords:** Diseases, Health care, Medical research, Risk factors

## Abstract

This study aimed to investigate national-level prevalence of COPD, proportion of patients diagnosed with and without COPD. We performed pulmonary function test (PFT) in 24,454 adults aged > 40 years for 8 years (2010–2017). The annual COPD prevalence increased from 13.1% in 2010 to 14.6% in 2012, followed by 13.3% in 2017. However, patients diagnosed with COPD ranged between 0.5 and 1.0% in the last 8 years, which means that only 5% of all COPD patients were diagnosed with COPD by doctors. We defined potential high-risk individuals as those with a FEV_1_/FVC ratio of < 0.70, who have not been diagnosed with COPD and other respiratory diseases tuberculosis, asthma, lung cancer. The proportion of this group was 80.8% in 2010 and 78.1% in 2017. The older age group, women, low-educated group, and current smokers who have been smoking for a long time are more likely to be in the high-risk group having a higher possibility to develop COPD but are not diagnosed with COPD appropriately. Although COPD prevalence was high in the ever, current, and heavy smokers, only the diagnosis rate of COPD in ever smokers was 2.38 times higher than never smokers, indicating that a system is needed to screen and intervention for these groups.

## Introduction

Chronic obstructive pulmonary disease (COPD) is the leading cause of morbidity and mortality worldwide. COPD accounts for more than half of health expenditures spent for respiratory disease (Forum of International Respiratory Societies. The Global Impact of Respiratory Disease. 2nd ed. Sheffield: European Respiratory Society, Forum of International Respiratory Societies; 2017). COPD is the third leading cause of death in the United States^1^, and WHO predicts that COPD will account for the third largest cause of death in 2030^2^. The prevalence of COPD continues to increase in the twenty-first century; it will be particularly severe in Asia owing to air pollution^3^. Prevalence of COPD is affected by methods, definition, and survey populations. Estimated COPD prevalence in the Asia Pacific region was 6.3%, but the WHO reported it to be 3.9%^4^. In the systematic review, COPD prevalence was estimated to be 7.8–14.1%, and as per the Western Pacific Regional Office of WHO, it was 10%. To accurately determine COPD prevalence, clinically proven tools such as pulmonary function tests (PFT) are needed. In a few countries, in approximately 10% patients, COPD was measured by actual airflow obstruction^5^.

Underdiagnosis of COPD is a public health concern^6^. COPD prevalence in South Korea in 2015 ranged from 13.1 to 14.6%^7^. Although Korea achieved universal health coverage via the mandatory National Health Insurance, among those with COPD as determined by spirometry, only 2.8% were diagnosed as COPD by physicians and the treatment rate was 1.6%^8^. Perhaps in countries without universal health insurance or in developing countries, the problem of underdiagnosis is higher^9^. COPD will be underestimated, but there is insufficient research on how underestimated it is. Despite being COPD patients, many individuals are unaware of their illness, and this group is a major public health threat. Therefore, these groups should be considered as potential risk groups. Although there are numerous studies related to COPD, the majority of them are based on small sample sizes or are limited to national studies that only measure prevalence through spirometry^10^; therefore, larger-scale studies are still needed.

Identifying the socioeconomic group with high COPD prevalence and determining those who are unaware of COPD are important. To decrease the rate of underdiagnosed COPD and to plan strategies for efficient screening for disease management, we investigated the national-level prevalence of COPD by airflow obstruction. First, we confirmed the trend of COPD prevalence by year by PFT. Second, we determined the proportion of patients diagnosed by doctors and those who were unaware of their COPD. Lastly, we analyzed the factors associated with COPD prevalence and potential high-risk group characteristics.

## Methods

### Study design and population

**Figure 1:**
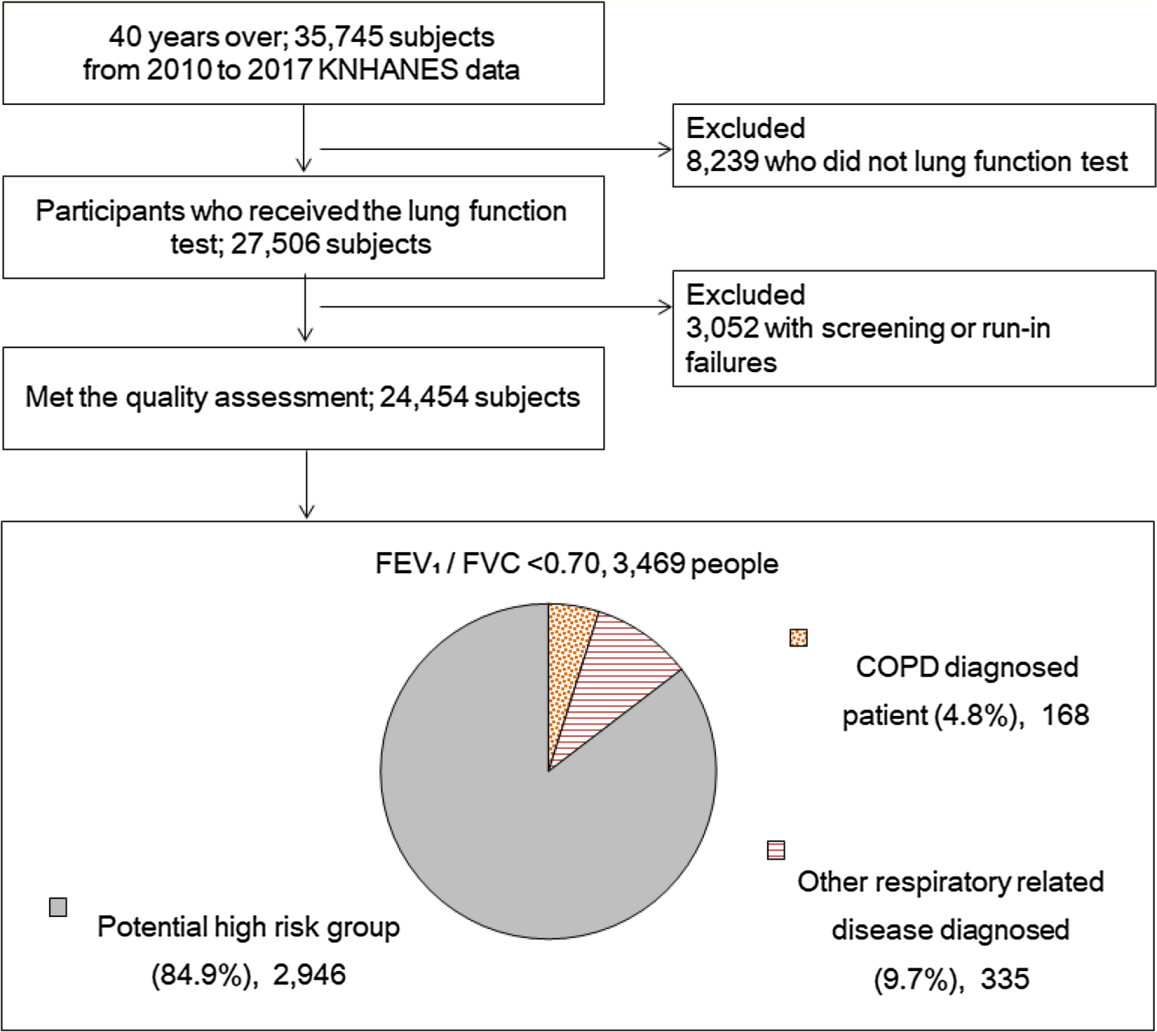
Flow diagram for study subject. *COPD* chronic obstructive pulmonary disease, *KNHANES* Korea National Health and Nutrition Health Examination Survey, *FEV*_*1*_ forced expiratory volume in one second, *FVC* forced vital capacity.

We used the Korea National Health and Nutrition Health Examination Survey (KNHANES) from 2010 to 2017. KNHANES surveys about 10,000 people each year by stratified multistage sampling design to show the representativeness of national indicators. The survey is conducted in three areas: health surveys like smoking and drinking; nutrition surveys like diet and nutrition; and physical examinations like measurement of height, weight, blood, and urine and PFT. We performed PFT in adults aged > 40 years, based on 8 years of data. Of the 35,745 individuals aged > 40 years, 24,454 were selected as the study subjects, excluding 8239 who did not receive a PFT and 3052 with screening or run-in failures despite receiving PFT. The definition of ‘COPD diagnosis’ is based on spirometric screening for the all participants. Via consensus and validation for the decades, the Global Initiative for Chronic Obstructive Lung Disease (GOLD) defined COPD by a fixed forced expiratory volume in 1 s (FEV_1_)/forced vital capacity (FVC) of 0.70, in 2001^11^. We included (1) those with FEV_1_/FVC of < 0.70 by GOLD standard defined as the COPD prevalence group and (2) group of patients diagnosed with COPD by doctors. (3) We defined potential high-risk among those with a FEV_1_/FVC ratio of < 0.70 who have not been diagnosed with COPD and other respiratory-related diseases such as TB, asthma, lung cancer. This is because the subjects who are not diagnosed with other respiratory diseases are likely to be unmanaged despite having COPD. Of the 24,454 participants, 3469 were classified into the COPD prevalence groups; of these, 168 were diagnosed with COPD by doctors and 335 were diagnosed with other respiratory-related diseases. The 2946 were classified as the potential high-risk group (Fig. 1).

### Measurement

The survey data and biological samples were collected by trained staff in two mobile clinic vehicles. The first mobile clinic was designed for physical examinations like measuring lung function. PFT was performed using the Dry rolling seal spiro, and then changed to the Vyntus spiro from June 2016. The test was performed by a trained professional, and after daily inspection, the analysis was conducted by the researcher in charge of the quality assessment on the day of the examination according to the refined ABCD assessment tool of GOLD guidelines^12^. PFT (V_max_ Model 2130; SensorMedics, Yorba Linda, CA, USA) was performed and assessed on the basis of the criteria of the American Thoracic Society and European Respiratory Society. Participants were then moved to the second mobile clinic to receive a health questionnaire. The survey was conducted face-to-face, and the responses are stored real time on tablet PC by the researcher. About 1 week after the two-step mobile clinic survey was completed, a nutritionist visited their households to conduct a nutrition survey.

### Variables

PFT measured FEV_1_ and FVC. FEV_1_/FVC ratio of < 0.70 was defined as COPD. COPD is closely related to age, sex^10,13^, and socioeconomic status such as income, education, job status, and residential area^3,14^. We classified income into quartiles. Regarding job status, white collar comprised individuals working in customer service area, finance, medical professionals, and office workers; blue collar comprised those in agriculture, forestry, fishery, factory, and construction workers; and non-jobs comprised those currently without jobs, housewives, and students.

The regional area was classified into metro city, other urban city, and rural area. Regarding health behavior, we considered major variables that could affect respiratory health like current smoking status, pack year (PY) of cigarettes, high-risk drinking, and walking practice (Person who walked for 30 min at a time over the past week for 5 days)^3,15,16^. Smoking is a known cause of COPD^3,17,18^. Smokers who had smoked more than five packs (100 cigarettes) in their lifetime and who currently smoked were defined as current smokers, those smoking ≥ 20 cigarettes daily as heavy smokers, and those who had smoked in the past but did not smoke currently as ever smokers. If you smoke one pack (20 cigarettes) daily for 10 years, you get 10 PY. Ever smoker will get 20 PY if a smoker has smoked a pack daily for the past 20 years. We divided PY into quartiles as follows: 1Q < 8 PY, 2Q < 20 PY or less, 3Q < 32 PY or less, and 4Q ≥ 32 PY. Next, the heavy drinkers in men were defined as those who drank more than seven drinks twice a week and in women as those who drank more than five drinks twice a week. Walking practice was defined as a person who walked for 30 min at a time over the past week for 5 days. COPD is associated with various health outcomes^19^. We defined patients with hypertension, diabetes, and dyslipidemia as those with non-communicable diseases (NCD) who had been diagnosed by doctors or were currently taking medication for these diseases^20^, and experience of cancer (stomach, liver, colon, breast, cervix, etc.) patient^21^.

### Statistical analysis

We showed the prevalence by year according to the COPD classification and proportion of patients with COPD, those with other respiratory diseases (TB, asthma, and lung cancer), and the potential high-risk group. Demographic analysis was conducted by dividing the patients into groups diagnosed by doctor and potential high-risk group. We performed multivariable logistic regression analysis by adjusting dependent variable to determine if each factor was related to COPD. All statistical analyses were weighted to reflect national representativeness according to the stratified multistage sampling design. All analyses were conducted using SAS for Windows 9.4.

### Ethics approval and consent to participate

The Institutional Review Board of the Korea Disease Control and Prevention Agency (KDCA) reviewed and approved the KNHANES (IRB Nos. 2010-02CON-21-C, 2011-02CON-06-C, 2012-01EXP-01-2C, 2013-07CON-03-4C, 2013-12EXP-03-5, 2018-01-03-P-A). All participants were informed about the purpose and provided written consent at the beginning of this investigation. This study was conducted in accordance with the Declaration of Helsinki, and all of the materials used in the article were only publicly available data.

## Results

### COPD prevalence and proportion of potential high-risk group

**Figure 2:**
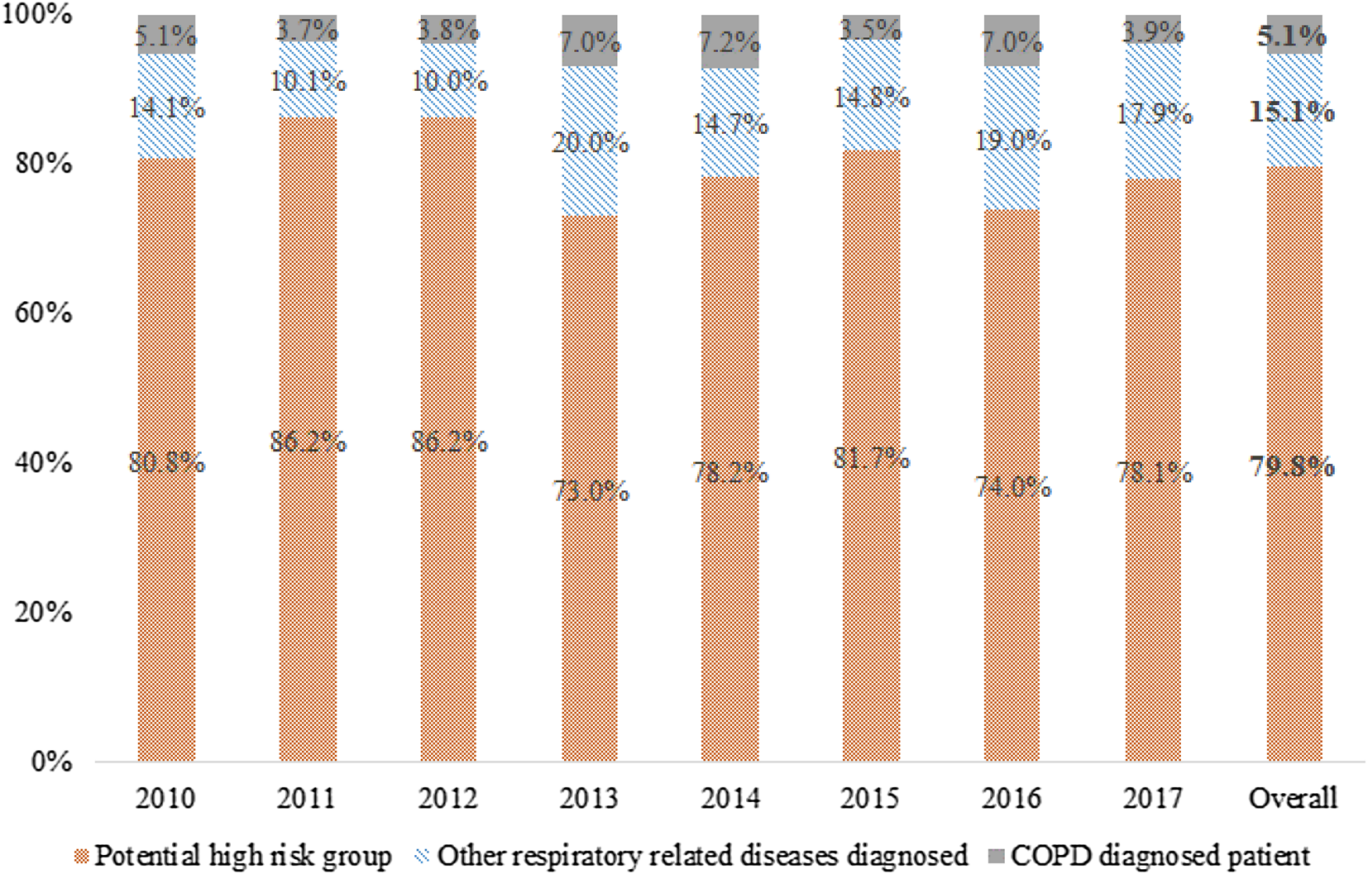
Proportion of patients diagnosed with chronic obstructive pulmonary disease (COPD) and diagnosed with other respiratory-related diseases (tuberculosis, asthma, and lung cancer) diagnosed among COPD prevalence.

The annual COPD prevalence increased from 13.1% in 2010 to 14.6% in 2012 followed by 13.3% in 2017. The prevalence of COPD diagnosed patients was 0.7% in 2010 and remained at 0.5–1.0% until 2017. The potential high-risk group for COPD was 11.7% in 2010 followed by 11.7% in 2017 after a slight increase and decrease (Table 1). In the COPD prevalence group, the number of patients diagnosed with COPD by doctors was 5.1% in 2010 followed by 3.9% in 2017 after a slight increase/decrease. The other respiratory-related disease diagnosed group that was not diagnosed with COPD comprised 10.0%–20.0%. The proportion of potential high-risk group that was not diagnosed with COPD and other respiratory diseases was 80.8% in 2010 and 78.1% in 2017 ranging between 73.0 and 86.2% (Fig. 2).

**Table 1:** Prevalence of COPD, diagnosed patient, and potential high-risk group in 2010–2017.

Year (participants)	COPD prevalence group^1^		COPD diagnosed patients		Potential high-risk (unrecognized) group^2^	
	Unweighted no. (weighted no.)^3^	Weighted %	Unweighted no. (weighted no.)	Weighted %	Unweighted no (weighted no.)*	Weighted %
Overall	3469 (3,031,257)	13.6	184 (173,007)	0.7	2946 (2,571,992)	12.3
2010 (2855)	347 (328,513)	13.1	15 (18,985)	0.7	302 (317,436)	11.7
2011 (3101)	410 (346,069)	13.2	14 (14,543)	0.5	369 (350,141)	12.3
2012 (3325)	514 (393,556)	14.6	19 (16,702)	0.6	461 (398,533)	13.6
2013 (2910)	409 (417,916)	13.5	27 (29,123)	0.9	329 (334,282)	11.7
2014 (2787)	424 (450,261)	14.2	25 (32,231)	1.0	364 (384,199)	13.0
2015 (2880)	406 (433,619)	13.4	19 (15,075)	0.5	342 (369,497)	12.3
2016 (3260)	480 (454,911)	13.6	29 (31,823)	1.0	389 (368,666)	11.9
2017 (3469)	479 (451,802)	13.3	20 (17,833)	0.5	390 (370,738)	11.7

*COPD* chronic obstructive pulmonary disease, *FEV*_*1*_ forced expiratory volume in 1 s, *FVC* forced vital capacity.

^1^Participants with FEV_1_/FVC < 0.70.

^2^Excluded those diagnosed with COPD, tuberculosis, asthma, and lung cancer in COPD prevalence.

^3^Estimated number of people reflecting actual population.

### Demographic analysis

The subjects of this study were 24,454 individuals who received lung function tests. Among them, 41.3% were aged 50–59 years, 34.0% were aged 40–49 years, 14.2% were aged 70–79 years, 9.2% were aged 60–69 years, and 1.3% were aged ≥ 80 years. By gender, 51.9% of men and income were in the fourth quarter, which was similar. In terms of education level, the percentage of individuals with high school education was the highest (33.5%), followed by the above college. Occupation was slightly higher in non-job (35.8%) than white collar (33.2%) and blue (31.0%), and in residential area, city, metro city, and rural areas were in order. Regarding smoking status, never smoker was the most common with 56.0%, followed by ever smoker (23.8%), current smoker (11.0), and heavy smoker (9.2%). Among those who had smoking experience, 2Q (more than 8PY and less than 20PY) was the most frequent in smoking pack year, and 11.4% of all participants were heavy drinkers. The walking practice rate was 37.8%. Patients with non-communicable disease who were diagnosed with at least hypertension, diabetes mellitus, or dyslipidemia accounted for 37.7% and those diagnosed with cancer accounted for 7.7%.

The potential high-risk group comprised those aged > 80 years, higher prevalence in the group 4Q, male, with low income, elementary, non-job, ever smoker, and PY of cigarettes, the group with NCDs, and with high prevalence of cancer diagnosis. There was statistical significance. However, there was no statistical significance between heavy drinkers and walking practices. Among COPD diagnosed patients, those in their 1970s had the highest rate of diagnosis. The diagnosis rates were high in the group with male, non-jobs, ever smoker, and high smoking PY, and the group with NCD, which showed a similar tendency except for age in comparison to the potential high-risk group (Supplement Table S1).

### Factors affecting COPD prevalence

Multivariable logistic regression showed that ever smokers in the COPD diagnosed patient group had a 2.38 times higher rate of diagnosis than never smokers. Furthermore, the group with PY of 4Q had a 2.81 times higher diagnosis rate than that of 1Q. The potential high-risk group had a higher prevalence of COPD in the 1950s (3.12), 1960s (9.04), 1970s (14.84), and those > 80 years (15.44) than those in the 1940s. The prevalence in males was 3.22 times higher than that in females in middle school graduates (1.28) and it was lower in elementary school graduates (1.42) than that in college graduates. The ever smokers had 1.32 times higher COPD prevalence, the current smokers had 2.22 times higher prevalence, and the heavy smokers had 2.31 times higher prevalence than the never smokers. Regarding PY, 3Q was 1.28 times higher and 4Q was 1.64 times higher than 1Q (Table 2).

**Table 2:** Multivariable logistic regression to determine the factors identifying subjects with COPD.

Overall	COPD diagnosed patients		Potential high-risk group	
	OR	95% CI	OR	95% CI
Age (years)				
40–49	Ref		Ref	
50–59	1.09	0.58–2.07	**3.12**	**2.45–3.98**
60–69	1.90	0.80–4.54	**9.04**	**6.75–12.10**
70–79	1.81	0.78–4.17	**14.84**	**11.21–19.63**
> 80	0.71	0.09–5.91	**15.44**	**9.37–25.44**
Sex				
Female	Ref		Ref	
Male	0.84	0.36–1.97	**3.22**	**2.60–3.99**
Income quartile				
Low	Ref		Ref	
Middle low	0.80	0.43–1.46	1.13	0.93–1.37
Middle high	0.67	0.38–1.20	0.92	0.75–1.13
High	0.57	0.29–1.12	1.20	0.98–1.48
Education				
Above college	Ref		Ref	
High school	0.96	0.49–1.88	1.21	0.99–1.47
Middle school	1.18	0.56–2.46	**1.28**	**1.01–1.62**
Elementary	0.72	0.36–1.61	**1.42**	**1.14–1.78**
Occupation				
White	Ref		Ref	
Blue	1.11	0.55–2.46	1.03	0.85–1.25
Non-jobs	2.09	0.99–4.38	1.10	0.90–1.35
Residential area				
Metro	Ref		Ref	
Other urban city	0.80	0.48–1.34	0.98	0.83–1.15
Rural area	1.34	0.73–2.46	1.10	0.91–1.32
Smoking status				
Never smoker	Ref		Ref	
Ever smoker	**2.38**	**1.04–5.47**	**1.32**	**1.03–1.69**
Current smoker	1.27	0.49–3.26	**2.22**	**1.70–2.89**
Heavy smoker	1.35	0.35–5.23	**2.31**	**1.73–3.07**
Smoking Pack year				
1Q (~ 8 PY)	Ref		Ref	
2Q (~ 20 PY)	1.33	0.61–2.92	1.02	0.81–1.27
3Q (~ 31.9 PY)	1.66	0.71–3.89	**1.28**	**1.00–1.64**
4Q (32 PY ~)	**2.81**	**1.33–5.92**	**1.64**	**1.73–3.07**
Heavy drinker				
No	Ref		Ref	
Yes	0.53	0.21–1.31	0.87	0.71–1.07
Walking^3^				
No	Ref		Ref	
Yes	0.92	0.59–1.44	1.02	0.90–1.16
NCD^4^				
No	Ref		Ref	
Yes	1.17	0.72–1.88	0.88	0.77–1.00
Cancer^5^				
No	Ref		Ref	
Yes	0.50	0.18–1.41	0.98	0.76–1.27

Significant values are in [bold].

^1^Unweighted number of respondents.

^2^Weighted percentage.

^3^Heavy drinker is consuming 5 drinks or more per week for women and 8 or more for men.

^4^Walking practice: Person who walked for 30 min at a time over the past week for 5 days.

^5^NCD: Non-Communicable Disease (physician diagnosis about hypertension, diabetes mellitus, dyslipidemia, etc.).

^6^Cancer: Experience of cancer (stomach, liver, colon, breast, cervix, etc.) patient.

## Discussion

In this study, patients diagnosed with COPD ranged between 0.5 and 1.0% in the last 8 years. However, those with COPD as per PFTs ranged from 13.1 to 14.6%, which was 10–20 times more than the actual number of patients diagnosed, which means that only 5% of all COPD patients were diagnosed with COPD by doctors. Especially the older age group, women, low-educated group, and current smokers who have been smoking for a long time are more likely to be in the high-risk group having a higher possibility to develop COPD but are not diagnosed with COPD appropriately. Although, only the diagnosis rate of COPD in ever smokers was 2.38 times higher than never smokers, indicating that a system is needed to screen and intervention for these groups.

As it is difficult to calculate COPD prevalence using PFT, various studies have estimated COPD prevalence. We showed a higher level than the existing predicted prevalence^4,10,22^. The study conducted by John^23^ estimated COPD prevalence in 12 countries using a mathematical model, and the prevalence in Korea was 6.2% at that time. As the year was different, it was difficult to directly compare it with the present study. The actual prevalence rate in 2010, which is the nearest year, was 13.1% that was about twice as high than expected, i.e., the number of actual COPD patients is likely to be higher than currently predicted. In other words, the burden of diseases caused by COPD worldwide is like to be higher than current forecasts. In our study, patients diagnosed with COPD ranged between 0.5 and 1.0% in the last 8 years. However, those with COPD as per PFTs ranged from 13.1 to 14.6%, which was 10–20 times more than the actual number of patients diagnosed, i.e., only 1/20 of all COPD patients were diagnosed with COPD by doctors. Excluding those diagnosed with respiratory diseases like TB, asthma, and lung cancer, eight in ten patients with COPD were likely to be unaware of COPD and other respiratory-related disease and to remain as the potential high-risk group without management or intervention. COPD is a chronic respiratory disease that needs to be managed. Airflow obstruction increases coronary events and mortality^17,24^ and contributes to deaths caused by respiratory diseases such as pneumonia^25^. Early COPD means early age onset (< 50 years) of COPD, whereas mild COPD represents mild airflow limitation (FEV_1_ ≥ 80% predicted)^26^. Early COPD accounts for 15% of COPD. However, its prognosis was poor with hazard ratio of 6.42 (95% CI 3.39–12.2) for hospitalization and 1.79 (1.28–2.52) for all-cause mortality^27^. Mild COPD causes accelerated FEV_1_ decline and increases mortality risk compared with that in those without COPD^26^. Although, early and mild COPD patients have substantial disease burden, their treatment strategy was limited because they are likely to be undiagnosed, thus not included in clinical research. Therefore, active COPD finding is recommended in patients with respiratory symptoms and/or risk factors^28^.

To prevent severe conditions and complications and to lower respiratory mortality, COPD should be detected at an early stage, managed^29^, and prioritized groups should be selected. Age, gender, and education are the most important factors related to COPD^10,13^. In our study, COPD prevalence was higher in older age groups, in men than in women, and at lower education levels. Particularly, those in their 1970s and those aged ≥ 80 years were about 15 times more likely to develop COPD than in their 1940s, men were three times more likely to develop COPD than women, and elementary school graduates were 1.5 times more likely to develop COPD than college graduates. However, patients with COPD were not statistically significant in terms of age; they have a high prevalence and are unlikely to be detected at an early stage and to be managed.

Although COPD prevalence was high in the ever, current, and heavy smokers, only the diagnosis rate of COPD in ever smokers was higher than never smokers, indicating that smoking is the most important cause of COPD^30^. This means that current and heavy smokers with COPD will continue to smoke unless any clinical or health issues is detected. COPD diagnosis provides the motivation to quit smoking^31^. Therefore, only ever smokers have a high diagnosis rate because smokers may have quit smoking after being diagnosed with COPD. However, in our study, most smokers were unaware that they had COPD and continued smoking. PY is the most powerful predictor for COPD^32^. In our study, the higher the PY, the higher the prevalence, and the 4Q group was diagnosed with COPD 2.8 times more than the 1Q group. As the lungs are irreversible once their functions are impaired, medical practitioners should encourage COPD patients who smoke to quit smoking^33^. However, this intervention is not easy because most patients with COPD are not detected.

In summary, social backgrounds may vary depending on countries and cultures, but previous studies^3,17,34,35^, and our studies have confirmed that the low socioeconomic group has a high prevalence of COPD. Therefore, when it is difficult to identify the COPD prevalence group, it would be universally valid to prioritize such low socioeconomic groups and smokers group. It has been proven that the older age group, women, low-educated group, and current smokers who have smoked for a long time are more likely to develop COPD. However, we found that these high-risk groups are not diagnosed with COPD in spite of being more likely to develop COPD. Early detection and management of COPD are necessary for personal health and for lowering the social burden of medical expenses^36^. The burden of medical expenses for COPD is a key challenge globally^3,37^, and the cost of medical treatment for COPD is rapidly increasing in Korea^38,39^. Therefore, there must be intervention from the public health viewpoint. The study by Kylie^6^ argued that screening in primary care needs to be further expanded since COPD is underdiagnosed. Accordingly, countries with health insurance as social insurance should actively consider including PFT in the national benefit services. Moreover, trainings according to clinical guidelines are necessary because primary care practitioners do not have sufficient knowledge and skills to diagnose and treat COPD^40^, and there is a need to strengthen the competencies of the primary medical-oriented COPD management system^35^.

COPD, a public health threat since 2000s, has been claimed to be intervened and managed^4^. The management of respiratory diseases like COPD will become more important in the future^41^. Nevertheless, there is still no important consensus. The results of our study further consolidated the previous evidence that COPD could be a significant threat to health problems and highlighted the need for further studies.

## Strength and limitations

According to the systematic review, while there are numerous studies related to COPD, only 0.3% of them have used spirometry to measure COPD, and population-based studies may have even fewer^10^. Therefore, our study has the advantage of using large-scale clinical data from 25,000 individuals assessed by a reliable diagnostic method, which can be considered as actual clinical evidence. While COPD should be diagnosed and managed, it is difficult to find studies that focus on identifying hidden high-risk groups that remain unmanaged. Under these circumstances, our study is among the first to identify the size and characteristics of the potential high-risk population. This study also has several limitations. First, as a cross-sectional study, it only shows the association with each variable and does not explain the causal relationship. When smokers are diagnosed with COPD, it is not possible to examine whether they quit smoking. Second, fine dust and indoor air pollution recently increased the incidence of COPD^42,43^, but factors related to environmental pollution were not controlled in our study. Therefore, studies that consider environmental factors are needed in the future.

## Conclusions

It has been proven that the older age group, women, low-educated group, and current smokers who have smoked for a long time are more likely to develop COPD. However, we found that these high-risk groups are not diagnosed with COPD despite being more likely to develop COPD.

## Supplementary Information

Supplementary Table S1.

## Data Availability

All materials used in the article were only publicly available data. Moreover, all of those data are non-identifying data, and anyone can use it. Data can be downloaded with permission from the KDCA KNHANEs website (https://knhanes.kdca.go.kr/knhanes/eng/). If you need the processed data, please contact the author to request the data.

## Abbreviations

COPD: Chronic obstructive pulmonary disease
GOLD: Global initiative for obstructive lung disease
FEV_1_: Forced expiratory volume in 1 s (FEV1)
FVC: Forced vital capacity
KDCA: Korea Disease Control and Prevention Agency
KNHANES: Korea National Health and Nutrition Health Examination Survey
NCD: Non-communicable diseases
PFT: Pulmonary function tests
PY: Pack year
TB: Tuberculosis

## References

[1] Leng S (2017). Dietary nutrients associated with preservation of lung function in Hispanic and non-Hispanic white smokers from New Mexico. Int. J. Chron. Obstruct. Pulmon. Dis..

[2] Alwan A. Global Status Report on Non-Communicable Diseases. WHO (2010).

[3] López-Campos JL, Tan W, Soriano JB (2016). Global burden of COPD. Respirology.

[4] Salvi SS, Manap R, Beasley R (2012). Understanding the true burden of COPD: The epidemiological challenges. Prim. Care Respir. J..

[5] Buist AS (2007). International variation in the prevalence of COPD (the BOLD Study): A population-based prevalence study. Lancet.

[6] Hill K (2010). Prevalence and underdiagnosis of chronic obstructive pulmonary disease among patients at risk in primary care. CMAJ.

[7] An TJ, Yoon HK (2018). Prevalence and socioeconomic burden of chronic obstructive pulmonary disease. J. Korean Med. Assoc..

[8] Hwang YI, Park YB, Yoo KH (2017). Recent trends in the prevalence of chronic obstructive pulmonary disease in Korea. Tuberculosis Respir. Diseases.

[9] Ranabhat CL, Atkinson J, Park M-B, Kim C-B, Jakovljevic M (2018). The influence of universal health coverage on life expectancy at birth (LEAB) and healthy life expectancy (HALE): A multi-country cross-sectional study. Front. Pharmacol..

[10] Adeloye, D. *et al.* Global and regional estimates of COPD prevalence: Systematic review and meta–analysis. *J. Glob. Health*. **5** (2015). 10.7189/jogh.05-020415 PMC4693508 26755942

[11] GOLD. Global initiative for chronic obstructive lung disease. (2019).

[12] Disease, G. I. f. C. O. L. (Global Initiative for Chronic Obstructive Lung Disease Inc, 2017).

[13] Ntritsos G (2018). Gender-specific estimates of COPD prevalence: A systematic review and meta-analysis. Int. J. Chron. Obstruct. Pulmon. Dis..

[14] Croft JB (2018). Urban-rural county and state differences in chronic obstructive pulmonary disease—United States, 2015. Morb. Mortal. Wkly Rep..

[15] Gershon AS (2015). Quantifying comorbidity in individuals with COPD: A population study. Eur. Respir. J..

[16] Cosio BG (2016). Defining the asthma-COPD overlap syndrome in a COPD cohort. Chest.

[17] Kurmi OP (2018). Excess risk of major vascular diseases associated with airflow obstruction: A 9-year prospective study of 05 million Chinese adults. Int. J. Chronic Obstruct. Pulmonary Disease..

[18] Kaluza J, Harris HR, Linden A, Wolk A (2019). Alcohol consumption and risk of chronic obstructive pulmonary disease: A prospective cohort study of men. Am. J. Epidemiol..

[19] Mannino DM, Thorn D, Swensen A, Holguin F (2008). Prevalence and outcomes of diabetes, hypertension and cardiovascular disease in COPD. Eur. Respir. J..

[20] Hillas G, Perlikos F, Tsiligianni I, Tzanakis N (2015). Managing comorbidities in COPD. Int. J. Chron. Obstruct. Pulmon. Dis..

[21] Young RP (2009). COPD prevalence is increased in lung cancer, independent of age, sex and smoking history. Eur. Respir. J..

[22] Halbert R (2006). Global burden of COPD: Systematic review and meta-analysis. Eur. Respir. J..

[23] Peabody JW (2005). COPD: A prevalence estimation model. Respirology.

[24] Duong M (2019). Mortality and cardiovascular and respiratory morbidity in individuals with impaired FEV1 (PURE): An international, community-based cohort study. Lancet Glob. Health.

[25] Restrepo MI, Mortensen EM, Pugh JA, Anzueto A (2006). COPD is associated with increased mortality in patients with community-acquired pneumonia. Eur. Respir. J..

[26] Singh D, D’Urzo AD, Donohue JF, Kerwin EM (2019). Weighing the evidence for pharmacological treatment interventions in mild COPD; a narrative perspective. Respir. Res..

[27] Colak Y, Afzal S, Nordestgaard B, Vestbo J, Lange P (2020). Prevalence, characteristics, and prognosis of early chronic obstructive pulmonary disease. The Copenhagen General Population Study. Am. J. Respir. Crit. Care Med..

[28] Hurd SS, Pauwels R (2002). Global initiative for chronic obstructive lung diseases (GOLD). Pulm. Pharmacol. Ther..

[29] Bellamy D, Smith J (2007). Role of primary care in early diagnosis and effective management of COPD. Int. J. Clin. Pract..

[30] Yoo KH (2011). Prevalence of chronic obstructive pulmonary disease in Korea: The fourth Korean National Health and Nutrition Examination Survey, 2008. Respirology.

[31] Kaplan, A. & Thomas, M. Screening for COPD: The gap between logic and evidence. *Eur. Respir. Rev*. **26** (2017). 10.1183/16000617.0113-2016 PMC9489098 28298389

[32] Burney P, Jarvis D, Perez-Padilla R (2015). The global burden of chronic respiratory disease in adults. Int. J. Tuberc. Lung Dis..

[33] López-Campos JL, Soler-Cataluña JJ, Miravitlles M (2020). Global strategy for the diagnosis, management, and prevention of chronic obstructive lung disease 2019 report: Future challenges. Arch. Bronconeumol..

[34] Yin P, Zhang M, Li Y, Jiang Y, Zhao W (2011). Prevalence of COPD and its association with socioeconomic status in China: Findings from China Chronic Disease Risk Factor Surveillance 2007. BMC Public Health.

[35] Herrera, A. C. *et al.* COPD underdiagnosis and misdiagnosis in a high-risk primary care population in four Latin American countries. A key to enhance disease diagnosis: The PUMA study. *PloS One*. **11** (2016). 10.1371/journal.pone.0152266 PMC4830516 27073880

[36] Lee YS (2019). The economic effect of early management in patients with early chronic obstructive pulmonary disease: Results from a population-based nationwide survey. Lung.

[37] Khakban A (2015). Ten-year trends in direct costs of COPD: A population-based study. Chest.

[38] Kim J (2013). The health care burden of high grade chronic obstructive pulmonary disease in Korea: Analysis of the Korean Health Insurance Review and Assessment Service data. Int. J. Chron. Obstruct. Pulmon. Dis..

[39] Yoo K-H (2016). Burden of respiratory disease in Korea: An observational study on allergic rhinitis, asthma, COPD, and rhinosinusitis. Allergy Asthma Immunol. Res..

[40] Lee J, Lee JH, Kim J-A, Rhee CK (2017). Trend of cost and utilization of COPD medication in Korea. Int. J. Chron. Obstruct. Pulmon. Dis..

[41] GHanizadeH, G., Heidari, M., Seifi, B., Jafari, H. & PakJouei, S. The effect of climate change on cardiopulmonary disease—A systematic review. *J. Clin. Diagnostic Res*. **11** (2017).

[42] Li M-H (2016). Short-term exposure to ambient fine particulate matter increases hospitalizations and mortality in COPD: A systematic review and meta-analysis. Chest.

[43] Roy MP (2019). COPD and indoor air pollution. BMJ.

