# Supplementary material for: Most patients with COPD are unaware of their health threats and are not diagnosed: a national-level study using pulmonary function test
Source: Sci Rep. 2023 Apr 11;13:5893. doi: 10.1038/s41598-023-32485-9 (PMC10090160; doi:10.1038/s41598-023-32485-9)
Supplement: Supplementary file 1 — Supplementary Table S1. [file 41598_2023_32485_MOESM1_ESM.docx]

Supplement Table S1. General characteristics of the subjects by COPD classification

Unit: n^1)^, %^2)^

|  | **All subjects** | | | **COPD diagnosed patients** | | | **Potential high-risk group** | | |
| --- | --- | --- | --- | --- | --- | --- | --- | --- | --- |
|  | n | % | Chi-square | n | % | Chi-square | n | % | Chi-square |
| **Subject**  **(Population weighted no.)** | 24454  (22,282,254) | 100.0 |  | 168  (176,315) | 0.71 |  | 2946  (2,893,491) | 12.3 |  |
| **Age** | 24454(22,282,254) | | | 168(176,315) | | | 2946(2,893,491) | | |
| 40–49 | 6839 | 34.0 | <.0001 | 24 | 0.4 | <.0001 | 197 | 3.4 | <.0001 |
| 50–59 | 10738 | 41.3 |  | 61 | 0.6 |  | 1059 | 11.0 |  |
| 60–69 | 2828 | 9.2 |  | 26 | 1.1 |  | 580 | 22.9 |  |
| 70–79 | 3708 | 14.2 |  | 53 | 1.4 |  | 991 | 29.7 |  |
| 80 over | 341 | 1.3 |  | 4 | 1.2 |  | 119 | (38.3 |  |
| **SEX** | 24454(22,282,254) | | | 168(176,315) | | | 2946(2,893,491) | | |
| Female | 10672 | 48.1 | <.0001 | 52 | 0.5 | <.0001 | 754 | 5.7 | <.0001 |
| Male | 13782 | 51.9 |  | 116 | 1.0 |  | 2192 | 19.4 |  |
| **Income quartile** | 24264(22,087,956) | | | 167(175,247) | | | 2920(2,865,247) | | |
| Low | 5600 | 24.5 | 0.5677 | 42 | 0.7 | 0.7238 | 723 | 13.1 | 0.0453 |
| Middle low | 6088 | 25.4 |  | 38 | 0.6 |  | 716 | 12.3 |  |
| Middle high | 6239 | 25.3 |  | 39 | 0.7 |  | 781 | 12.5 |  |
| High | 6337 | 24.9 |  | 48 | 0.8 |  | 700 | 11.1 |  |
| **Education** | 23637(21,481,659) | | | 165(173,183) | | | 2837(2,778,552) | | |
| Above college | 5866 | 26.2 | <.0001 | 41 | 0.7 | 0.3923 | 450 | 7.2 | <.0001 |
| High school | 7573 | 33.5 |  | 47 | 0.6 |  | 772 | 10.1 |  |
| Middle school | 3499 | 14.4 |  | 31 | 0.9 |  | 482 | 14.6 |  |
| Elementary | 6699 | 25.9 |  | 46 | 0.8 |  | 1133 | 19.1 |  |
| **Occupation** | 23619(21,460,586) | | | 165(173,183) | | | 2835(2,774,651) | | |
| White | 7376 | 33.2 | <.0001 | 30 | 0.4 | <.0001 | 520 | 7.2 | <.0001 |
| Blue | 7062 | 31.0 |  | 39 | 0.6 |  | 1052 | 14.9 |  |
| Non-jobs | 9181 | 35.8 |  | 96 | 1.1 |  | 1263 | 14.7 |  |
| **Residential area** | 24454(22,282,254) | | | 168(176,315) | | | 2946(2,893,491) | | |
| Metro | 8699 | 34.8 | <.0001 | 77 | 0.8 | 0.0931 | 1273 | 11.6 | <.0001 |
| Other urban city | 11075 | 46.2 |  | 51 | 0.5 |  | 930 | 10.9 |  |
| Rural area | 4680 | 19.0 |  | 40 | 0.9 |  | 743 | 16.4 |  |
| **Smoking status** | 23888(21,721,032) | | | 163(171,604) | | | 2864(2,805,378) | | |
| Never smoker | 14290 | 56.0 | <.0001 | 54 | 0.4 | <.0001 | 895 | 6.4 | <.0001 |
| Ever smoker | 5511 | 23.8 |  | 73 | 1.3 |  | 1131 | 20.0 |  |
| Current smoker | 2306 | 11.0 |  | 21 | 0.7 |  | 463 | 18.9 |  |
| Heavy smoker | 1781 | 9.2 |  | 15 | 0.8 |  | 375 | 19.3 |  |
| **Smoking Pack year** | 9542(9,745,870) | | | 168(176,315) | | | 2964(2,893,491) | | |
| 1Q (~8 PY) | 2276 | 23.6 | <.0001 | 84 | 0.5 | <.0001 | 1445 | 8.3 | <.0001 |
| 2Q (~20 PY) | 2942 | 31.8 |  | 21 | 0.7 |  | 445 | 15.6 |  |
| 3Q (~31.9 PY) | 2072 | 22.2 |  | 21 | 1.1 |  | 391 | 20.6 |  |
| 4Q (32 PY ~) | 2252 | 22.4 |  | 42 | 2.0 |  | 665 | 33.3 |  |
| **Heavy drinker**^3)^ | 24454(22,282,254) | | | 168(176,315) | | | 2946(2,893,491) | | |
| No | 22608 | 88.6 |  | 156 | 0.7 | 0.3169 | 2622 | 12.2 | 0.2487 |
| Yes | 2386 | 11.4 |  | 12 | 0.5 |  | 324 | 13.1 |  |
| **Walking practice**^4)^ | 23604(21,450,823) | | | 162(169,911) | | | 2828(2,769,597) | | |
| No | 14768 | 62.2 |  | 96 | 0.7 | 0.6013 | 1711 | 12.0 | 0.2289 |
| Yes | 8836 | 37.8 |  | 66 | 0.8 |  | 1117 | 12.6 |  |
| **NCD**^5)^ | 24454(22,282,254) | | | 168(176,315) | | | 2946(2,893,491) | | |
| No | 14666 | 62.3 |  | 77 | 0.5 | 0.0007 | 1457 | 9.9 | <.0001 |
| Yes | 9788 | 37.7 |  | 91 | 1.0 |  | 1489 | 16.2 |  |
| **Cancer**^6)^ | 14991(14,171,681) | | | 120(125,562) | | | 1787(1,783,826) | | |
| No | 13691 | 92.3 |  | 112 | 0.8 | 0.5904 | 1605 | 11.8 | 0.0022 |
| Yes | 1300 | 7.7 |  | 8 | 0.6 |  | 182 | 15.3 |  |

^1)^ Unweighted number of respondents

^2)^ Weighted percentage

^3)^ Heavy drinker is consuming 5 drinks or more per week for women and 8 or more for men.

^4)^ Walking practice: Person who walked for 30 minutes at a time over the past week for 5 days.

^5)^ NCD: Non-Communicable Disease (physician diagnosis about hypertension, diabetes mellitus, dyslipidemia, etc.)

^6)^ Cancer: Experience of cancer (stomach, liver, colon, breast, cervix, etc.) patient
